# The role of PD-1 and PD-L1 in T-cell immune suppression in patients with hematological malignancies

**DOI:** 10.1186/1756-8722-6-74

**Published:** 2013-09-30

**Authors:** Li Shi, Shaohua Chen, Lijian Yang, Yangqiu Li

**Affiliations:** 1 Institute of Hematology, Jinan University, Guangzhou 510632, China; 2 Key Laboratory for Regenerative Medicine of Ministry of Education, Jinan University, Guangzhou 510632, China

## Abstract

T-cell activation and dysfunction relies on direct and modulated receptors. Based on their functional outcome, co-signaling molecules can be divided as co-stimulators and co-inhibitors, which positively and negatively control the priming, growth, differentiation and functional maturation of a T-cell response. We are beginning to understand the power of co-inhibitors in the context of lymphocyte homeostasis and the pathogenesis of leukemia, which involves several newly described co-inhibitory pathways, including the programmed death-1 (PD-1) and PD-1 ligand (PD-L1) pathway. The aim of this review is to summarize the PD-1 and PD-L1 biological functions and their alterative expression in hematological malignancies. The role of PD-1 and PD-L1 in T-cell immune suppression and the potential for immunotherapy via blocking PD-1 and PD-L1 in hematological malignancies are also reviewed.

## Introduction

Leukemia, particularly aggressive refractory hematological malignancies unresponsive to upfront therapy, remains a difficult condition to treat. The focus of therapy is to achieve complete disease remission. Therefore, alternative treatment options that utilize immunotherapy while minimizing toxicity are warranted [1-3]. It is well known that persistent immunodeficiency is a common feature in patients with leukemia. Moreover, T cell function becomes suppressed with disease progression. Such immune dysfunction may be due to a disorder in thymic output function (in particular in young patients), which results in a lower level of naive T-cells in the peripheral blood available for an immune response to the proliferation and abnormal expression of the T cell receptor (TCR) repertoire. This condition results in an impaired specific antigen response and abnormal TCR signal transduction, which results in lower T cell activation for an immune response [4-9]. Moreover, increasing data have shown that peripheral T-cell tolerance is an essential property of the specific immune response to tumor cells. T-cell anergy is defined as the state in which T-cells fail to respond to previously encountered antigenic stimulation by functional APCs. Such T-cells lose the ability to autonomously produce IL-2 [10]. In addition, the low cytotoxicity of T-cells may be related to the high expression level of inhibitory molecules including programmed death-1 (PD-1), LAG-3 and NKG2A in CD8+T cells [11]. Moreover, it has been shown that the PD-1 ligand (PD-L1) is highly expressed in leukemia cells. In addition, PD-1/PD-L1 interactions contribute to functional T-cell impairment, which fails to elicit minimal residual disease and may be related to leukemia relapse. This phenomenon may be one of the reasons why immature leukemic progenitor cells escape the immune system i.e., by inhibiting T-cell function via the PD-1/PD-L1 pathway [12].

### Structural features of PD-1 and PD-L1

**Figure 1:**
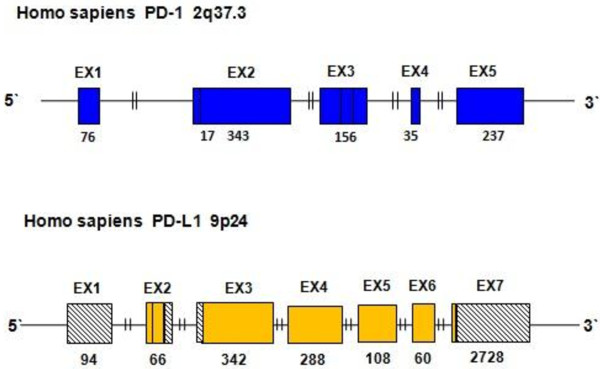
**Schematic structure genomic organization of PD-1 and PD-L1 gene.** The bars represent the exons (EX) and the lines represent introns. The blue bars are exons of the PD-1 gene which encode different regions of the PD-1 protein, including: L-region (EX1), hydrophilic-region (EX2), V-likedomain (EX2), connecting-region (EX3), transmembrane-region (EX3), C-like-domain (EX3), intracytoplasmic regions (EX4) and cytoplasmic-region (EX5) [15]. The yellow bars are exons of the PD-L1 gene which encode different regions of the PD-L1 protein, including: Immunoglobulin V-set domain (EX2),immunoglobulin constant domain (EX3), transmembrane region (EX4-EX7); diagonal line filled black and white bar represent the exons which do not express post transcriptional alternative splicing [NM_014143.3]. The numbers under the exons are the number of nucleotides corresponding to size of each exon.

The PD-1 gene is a CD28 family member that is a member of the immunoglobulin gene superfamily. Murine PD-1 mRNA expression has been shown to be correlated with activation-induced apoptosis in a mouse T-cell hybridoma cell line and murine thymocytes [13]. PD-1 is expressed on activated T cells, B cells, and myeloid cells. Human PD-1 is a homolog of murine PD-1 (mPD-1), which was originally isolated by Ishida et al. from apoptotic T-cell hybridomas by subtractive hybridization [14]. The human PD-1 gene is located on chromosome 2 at band q37, and the full length PD-1 cDNA is 2,106 nucleotides long and encodes a predicted protein of 288 amino acid residues. The human PD-1 and mPD-1 genes share 70% homology at the nucleotide level and 60% homology at the amino acid level. The PD-1 gene is encoded by five exons (EX1–EX5). EX1 (76 bp) encodes an L-region (25 aa) followed by an intron of 5,781 bp. EX2 (360 bp) encodes a short hydrophilic-region (6 aa) and an extracellular V-like domain (120 aa) and is separated from EX3 (156 bp) by an intron of 267 bp that encodes a connecting-region (23 aa), transmembrane-region (24 aa) and a portion of the C-like-domain (5 aa). The EX4 (35 bp) and EX5 intracytoplasmic regions (237 bp) are separated by an intron of 651 bp encoding most of the cytoplasmic-region (12 and 79 aa) (Figure 1) [15]. Five human PD-1 isoforms have been identified [16,17]. PD-1 is a 50–55 kDa type I transmembrane protein with a single IgV domain in the extracellular region. The cytoplasmic region of PD-1 contains an ITIM (immuno-receptor tyrosine-based inhibitory motif) and an ITSM (immuno-receptor tyrosine-based switch motif), and the latter is essential for the inhibitory function of TCR signaling [18]. PD-L1, which is also known as B7-H1 (CD274), is a cell surface protein of B7 family member. This protein is expressed on immune or non-hematopoietic cells. The human PD-L1 gene is located at 9p24, and the full length PD-L1 cDNA is 870 bp. The open reading frame of the PD-L1 gene encodes a putative type I transmembrane protein of 290 amino acids consisting of immunoglobulin V-like and C-like domains, a hydrophobic transmembrane domain and a cytoplasmic tail of 30 amino acids [19].

### Biological functions of PD-1 and PD-L1

As the main ligand for PD-1, PD-L1 induces a co-inhibitory signal in activated T-cells and promotes T-cell apoptosis, anergy and functional exhaustion [20,21]. T cell activation requires a TCR-mediated signal in addition to TCR signaling, and the strength and duration of T-cell activation are mainly determined by the net effect of positive and negative co-stimulation and cytokines from antigen-presenting cells (APCs). However, the ability to shape the outcome of positive vs. negative co-stimulation relies, at least partially, on the temporal and spatial expression of stimulatory and inhibitory ligands for co-signaling receptors [22]. Some molecular pairs attenuate the strength of the TCR signal, a process called co-inhibition [23,24]. The effect of the PD-1 and PD-L1 interaction is thought to be important for co-inhibition during the T-cell initiation of an immune response. T-cell activation also induces the expression of PD-1, while cytokines such as INF-γ and IL-4, which are produced after T-cell activation, up regulate PD-1 ligands, establishing a feedback loop that attenuates immune responses and limits the extent of immune-mediated tissue damage unless the activation is overridden by strong co-stimulatory signals. In proximity to the TCR signaling complex, PD-1 delivers a co-inhibitory signal upon binding with either of its two ligands PD-L1 or PD-L2. Ligand engagement results in the tyrosine phosphorylation of the PD-1 cytoplasmic domain and the recruitment of phosphatases, particularly SHP2 [25]. This recruitment results in dephosphorylation of the TCR proximal signaling molecules including ZAP70, PKCθ, and CD3ζ, leading to attenuation of the TCR/CD28 signal [26]. The ligation of PD-L1 on T-cells by either specific monoclonal antibodies or immobilized PD-1 co-stimulates T-cell growth and cytokine secretion [22].

### Expression characteristics of PD-1 and PD-L1

PD-1 is expressed in the thymus primarily on CD4^-^CD8^-^ (double negative) T-cells late in the transition from double negative to double positive cells. Double negative γδ thymocytes express high levels of PD-1, and natural killer T-cells express low levels of PD-1. Moreover, interesting findings have shown that PD-L1 is expressed at high levels by activated CD4+T cells [22]. Although PD-1 was not preferentially expressed in pro-B cells from human fetal bone marrow, treatment of isolated pro-B cells with IL-7 resulted in a dramatic increase in expression [16,17,27]. PD-L1 is expressed on almost all types of lymphohematopoietic cells at varying levels and is constitutively expressed on T-cells, B-cells, macrophages and dendritic cells (DCs). This ligand is further upregulated and strongly induced by mitogenic stimulation and IFN-γ, which is reminiscent of PD-1 receptor expression [28,29]. Moreover, the PD-L1 splice variant expression pattern was variable in different individuals and different cellular statuses. PD-L1 expression may be regulated at the posttranscriptional level through alternative splicing, and modulation of PD-L1 isoform expression may influence the outcome of specific immune responses in peripheral tissues [30]. The expression of PD-L1 is also detected on non-lymphoid cells e.g., endothelial cells in the heart, β cells in the pancreas, glial cells in inflamed brains and muscle cells [31-35]. Moreover, PD-L1 is abundant in human carcinomas of the lung, ovary and colon and in melanoma [36] and leukemic cells [28,37,38]. The latter finding appears to be important for cancer immunotherapy.

### Alterative expression of PD-1 in T cells from patients with hematological malignancies

Increasing data have shown that PD-1 is expressed at a higher level in T cells from tumor patients [39]. Recently, it was reported that CML-specific cytotoxic T-cells (CTLs) maintain only limited cytotoxic activity, do not produce interferon-γ or tumor necrosis factor-α, and do not expand after restimulation. These CTLs were characterized by the high expression of PD-1, and their target CML cells expressed higher levels of PD-L1 [38]. This phenomenon was not only found in a CML mouse model but also in patients with CML [40]. Thus, higher PD-1 expression in CTLs is related to inhibition of the effector phase of T-cell responses and reduced antitumor activity. It was more recently reported that PD-1 expression in CD4^+^ and CD8^+^ T-cells is significantly higher in patients with chronic lymphocytic leukemia (CLL), while the levels of PD-1 expression on both CD4+CD25+ and CD4+CD25- T-cells were increased in adult T-cell leukemia/lymphoma (ATL), but not in CD8+T cells [41-43]. More importantly, PD-1 was markedly elevated in tumor-infiltrating and peripheral T-cells from patients with Hodgkin’s lymphoma (HL) [33].

### The PD-L1 expression pattern in leukemia and lymphoma cells and the tumor microenvironment

A higher expression of PD-L1 was found in the majority of different hematological malignant cells, including primary mediastinal large B-cell lymphoma, T-cell/histiocyte-rich B-cell lymphoma, EBV-positive and EBV-negative PTLD, EBV-associated diffuse large B-cell lymphoma (DLBCL), plasmablastic lymphoma, extranodal NK/T-cell lymphoma, Burkitt’s lymphoma, HHV8-associated Kaposi sarcoma, B-cell leukemia, CML, AML cells, as well as in ATL cells in some patients [37,38,40,41,44,45]. PD-L1 expression was also demonstrated in primary Hodgkin/Reed-Sternberg (H/RS) cells [33]. Moreover, PD-L1 is highly expressed on tumor-infiltrating macrophages and the surface of tumor cells and antigen-presenting cells in the tumor microenvironment [30,31]. It appears to be clear that PD-L1/PD-1 co-stimulation has to be targeted in the tumor microenvironment. Tumor cells upregulate PD-L1 to dampen CTL attack. This upregulation is possibly a consequence of pro-inflammatory cytokine production by tumor infiltrating immune cells. For example, the IFN-γ produced by inflammatory cells acts as a potent PD-L1 up-regulator [46].

### PD-1 and PD-L1 relative immune suppression in hematological malignancies

Tumor-associated immune suppression can lead to defective T-cell-mediated antitumor immunity. Based on the finding that PD-L1 is up-regulated on HL cells, and PD-1 is markedly elevated in the tumor-infiltrating or peripheral T cells of HL patients, blockade of the PD-1 signaling pathway inhibits SHP-2 phosphorylation and restores the IFN-γ-producing function of HL-infiltrating T-cells [44]. According to these results, the deficient cellular immunity observed in HL patients may be explained as "T-cell exhaustion," which is led by the activation of PD-1-PD-L1 signaling pathway. This finding provides a potentially effective immunologic strategy for the treatment of HL [44]. Increasing data indicate that the PD-1-PD-L1 signaling pathway is related to immune suppression and disease progression. Zhou Q et al. demonstrated a unique phenotype i.e., co-expression of Tim-3 and PD-1 on CD8^+^ T cells increased during AML progression. Combined PD-1/PD-L1 and Tim-3/galectin-9 blockade could rescue mice from AML lethality [45], and this may be beneficial for preventing CD8^+^T-cell exhaustion in patients with hematological malignancies.

Moreover, data have shown cancer cell associated PD-L1 increases during the apoptosis of antigen-specific human T-cell clones *in vitro*[36]. In CML patients, T cells may be under the control of different immune escape mechanisms. CML with Sokal high risk have an increase in the number of myeloid-derived suppressor cells (MDSCs), which are an important immunosuppressive cell population in the tumor microenvironment. These MDSCs upregulate the expression of PD-L1/PD-1, arginase 1 and soluble CD25. While PD-L1 blockade does not increase T-cell proliferation, it does upregulate IL-2 secretion [47]. Therefore, combining anti-PD-1 therapy, which may re-educate MDSCs, to favor the recruitment of adoptively transferred, tumor-specific T-cells to lead to an improved antitumor response may be considered [48].

### The role of PD-1 and PD-L1 targeted immunotherapy in hematological malignancies

Recently, target immunotherapy using PD-1 and PD-L1 monoclonal antibodies (MoAbs) was demonstrated to significantly induce durable tumor regression and prolong disease stabilization in patients with selected advanced cancers, including non–small cell lung cancer, a tumor considered to be non-responsive to immunotherapy [39,49]. These results have led to many studies evaluating the effects of the target inhibition of PD-1 and PD-L1 in different cancers including hematological malignancies.

The findings of PD-1 and PD-L1 expression characteristics in leukemia and lymphomas with defective T-cell immune responses have implications for the design of T-cell-based cancer immunotherapy, and blockade of the PD-1/PD-L1 pathway may be a clinically effective strategy [36,41,50]. For example, an anti-PD-L1 blocking antibody boosted the proliferation and IFN-γ secretion of allogeneic T-cells responding to anaplastic large cell lymphoma (ALCL) and DLBCL cells. In autologous cultures of primary ALCL and DLBCL cells, PD-L1 blockade enhanced the secretion of the inflammatory cytokines IFN-γ, granulocyte macrophage colony-stimulating factor, IL-1, IL-6, IL-8, IL-13, TNF-α, and macrophage inflammatory protein-1α. In establishing cell lines from an aggressive PD-L1^+^mature B-cell lymphoma, it was also noted that PD-L1 expression could be lost under certain *in vitro* culture conditions [51]. Moreover, the phase I clinical study showed the CT-011 which is a humanized IgG1 monoclonal antibody against PD1 to be safe and well tolerated in patients with AML, CLL, non-Hodgkin’s lymphoma (NHL), HL or multiple myeloma (MM) at an advanced stage of their disease and following chemotherapy and/or stem cell transplantation. Clinical benefit was observed in 33% of the patients with one complete remission. The elevated peripheral blood CD4+, CD8+, and CD69+T cells were detected in CT-011 treated patients. However, no change in the levels of IFN-γ or t TNF-α was noted in sera derived from hematologic malignancy patients following treatment with CT-011. And a phase II clinical study evaluating the safety and efficacy of CT-011 administered at the dose level of 1.5 mg/kg in diffuse large B-cell lymphoma following autologous bone marrow transplantation has been initiated [52].

## Conclusions

The upregulation of PD-1 and PD-L1 is a common phenomenon in leukemia and lymphomas that leads to double T-cell immunodeficiency, low proliferation and activation effects, and higher immune suppression in patients. These findings further characterized the immune escape mechanisms and allowed understanding the lower effects of the T-cell immunotherapeutic efficacies in hematological malignancies. Moreover, the detection of PD-1 and PD-L1 may be considered a novel prognostic marker in these patients. Targeted inhibition of PD-1 and PD-L1 by different methods, such as MoAbs, small molecules or siRNAs, may significantly affect immunotherapeutic efficacies. Moreover, T-cell immunodeficiency in leukemia patients may be associated with different causes of immune suppression, such as alterative expression of TCR signaling pathway members and the immune negative feedback regulator A20 [5,53]. The PD-1 and PD-L1 interaction may reduce the apoptosis of tumor cells by suppressing T-cell function; thus, they may share a common signal transduction, and the development of a comprehensive strategy for special immunotherapy targeting this different pathway is needed.

## Competing interests

The authors declare that they have no competing interests.

## Authors’ contributions

The concept of this paper was devised by YQL, LS, SHC, LJY and YQL contributed to the intellectual input of the paper. All authors read and approved the final manuscript.

## References

[B1] Reagan JL Fast LD Safran H Nevola M Winer ES Castillo JJ Butera JN Quesenberry MI Young CT Quesenberry PJ Cellular immunotherapy for refractory hematological malignancies J Transl Med 2013 11 150 10.1186/1479-5876-11-150 23782682 PMC3689050

[B2] Lu K Wang X Therapeutic advancement of chronic lymphocytic leukemia J Hematol Oncol 2012 5 55 10.1186/1756-8722-5-55 22980425 PMC3465197

[B3] Takahashi S Downstream molecular pathways of FLT3 in the pathogenesis of acute myeloid leukemia: biology and therapeutic implications J Hematol Oncol 2011 4 13 10.1186/1756-8722-4-13 21453545 PMC3076284

[B4] Chen X Woiciechowsky A Raffegerst S Schendel D Kolb HJ Roskrow M Impaired expression of the CD3-zeta chain in peripheral blood T cells of patients with chronic myeloid leukaemia results in an increased susceptibility to apoptosis Br J Haematol 2000 111 3 817 825 11122143

[B5] Zha X Yan X Shen Q Zhang Y Wu X Chen S Li B Yang L Geng S Weng J Alternative expression of TCRzeta related genes in patients with chronic myeloid leukemia J Hematol Oncol 2012 5 74 10.1186/1756-8722-5-74 23228155 PMC3544630

[B6] Li Y Geng S Du X Chen S Yang L Wu X Li B Schmidt CA Przybylski GK Restricted TRBV repertoire in CD4+ and CD8+ T-cell subsets from CML patients Hematology 2011 16 1 43 49 10.1179/102453311X12902908411634 21269567

[B7] Li Y Geng S Yin Q Chen S Yang L Wu X Li B Du X Schmidt CA Przybylski GK Decreased level of recent thymic emigrants in CD4+ and CD8+T cells from CML patients J Transl Med 2010 8 47 10.1186/1479-5876-8-47 20470401 PMC2880023

[B8] Rezvany MR Jeddi-Tehrani M Osterborg A Kimby E Wigzell H Mellstedt H Oligoclonal TCRBV gene usage in B-cell chronic lymphocytic leukemia: major perturbations are preferentially seen within the CD4 T-cell subset Blood 1999 94 3 1063 1069 10419899

[B9] Torelli GF Paolini R Tatarelli C Soriani A Vitale A Guarini A Santoni A Foa R Defective expression of the T-cell receptor-CD3 zeta chain in T-cell acute lymphoblastic leukaemia Br J Haematol 2003 120 2 201 208 10.1046/j.1365-2141.2003.04044.x 12542476

[B10] Schwartz RH A cell culture model for T lymphocyte clonal anergy Science 1990 248 4961 1349 1356 10.1126/science.2113314 2113314

[B11] Bos R Marquardt KL Cheung J Sherman LA Functional differences between low- and high-affinity CD8(+) T cells in the tumor environment Oncoimmunology 2012 1 8 1239 1247 10.4161/onci.21285 23243587 PMC3518496

[B12] Norde WJ Maas F Hobo W Korman A Quigley M Kester MG Hebeda K Falkenburg JH Schaap N de Witte TM PD-1/PD-L1 interactions contribute to functional T-cell impairment in patients who relapse with cancer after allogeneic stem cell transplantation Cancer Res 2011 71 15 5111 5122 10.1158/0008-5472.CAN-11-0108 21659460

[B13] Vibhakar R Juan G Traganos F Darzynkiewicz Z Finger LR Activation-induced expression of human programmed death-1 gene in T-lymphocytes Exp Cell Res 1997 232 1 25 28 10.1006/excr.1997.3493 9141617

[B14] Ishida Y Agata Y Shibahara K Honjo T Induced expression of PD-1, a novel member of the immunoglobulin gene superfamily, upon programmed cell death EMBO J 1992 11 11 3887 3895 1396582 10.1002/j.1460-2075.1992.tb05481.x PMC556898

[B15] Garapati VP Lefranc MP IMGT Colliers de Perles and IgSF domain standardization for T cell costimulatory activatory (CD28, ICOS) and inhibitory (CTLA4, PDCD1 and BTLA) receptors Dev Comp Immunol 2007 31 10 1050 1072 10.1016/j.dci.2007.01.008 17391759

[B16] Finger LR Pu J Wasserman R Vibhakar R Louie E Hardy RR Burrows PD Billips LG The human PD-1 gene: complete cDNA, genomic organization, and developmentally regulated expression in B cell progenitors Gene 1997 197 1–2 177 187 9332365 10.1016/s0378-1119(97)00260-6

[B17] Shinohara T Taniwaki M Ishida Y Kawaichi M Honjo T Structure and chromosomal localization of the human PD-1 gene (PDCD1) Genomics 1994 23 3 704 706 10.1006/geno.1994.1562 7851902

[B18] Okazaki T Wang J PD-1/PD-L pathway and autoimmunity Autoimmunity 2005 38 5 353 357 10.1080/08916930500124072 16227150

[B19] Dong H Zhu G Tamada K Chen L B7-H1, a third member of the B7 family, co-stimulates T-cell proliferation and interleukin-10 secretion Nat Med 1999 5 12 1365 1369 10.1038/70932 10581077

[B20] Butte MJ Keir ME Phamduy TB Sharpe AH Freeman GJ Programmed death-1 ligand 1 interacts specifically with the B7-1 costimulatory molecule to inhibit T cell responses Immunity 2007 27 1 111 122 10.1016/j.immuni.2007.05.016 17629517 PMC2707944

[B21] Francisco LM Salinas VH Brown KE Vanguri VK Freeman GJ Kuchroo VK Sharpe AH PD-L1 regulates the development, maintenance, and function of induced regulatory T cells J Exp Med 2009 206 13 3015 3029 10.1084/jem.20090847 20008522 PMC2806460

[B22] Chen L Co-inhibitory molecules of the B7-CD28 family in the control of T-cell immunity Nat Rev Immunol 2004 4 5 336 347 10.1038/nri1349 15122199

[B23] Okazaki T Honjo T PD-1 and PD-1 ligands: from discovery to clinical application Int Immunol 2007 19 7 813 824 10.1093/intimm/dxm057 17606980

[B24] Keir ME Butte MJ Freeman GJ Sharpe AH PD-1 and its ligands in tolerance and immunity Annu Rev Immunol 2008 26 677 704 10.1146/annurev.immunol.26.021607.090331 18173375 PMC10637733

[B25] Freeman GJ Structures of PD-1 with its ligands: sideways and dancing cheek to cheek Proc Natl Acad Sci USA 2008 105 30 10275 10276 10.1073/pnas.0805459105 18650389 PMC2492504

[B26] Chemnitz JM Parry RV Nichols KE June CH Riley JL SHP-1 and SHP-2 associate with immunoreceptor tyrosine-based switch motif of programmed death 1 upon primary human T cell stimulation, but only receptor ligation prevents T cell activation J Immunol 2004 173 2 945 954 15240681 10.4049/jimmunol.173.2.945

[B27] Nishimura H Agata Y Kawasaki A Sato M Imamura S Minato N Yagita H Nakano T Honjo T Developmentally regulated expression of the PD-1 protein on the surface of double-negative (CD4-CD8-) thymocytes Int Immunol 1996 8 5 773 780 10.1093/intimm/8.5.773 8671666

[B28] Ishida M Iwai Y Tanaka Y Okazaki T Freeman GJ Minato N Honjo T Differential expression of PD-L1 and PD-L2, ligands for an inhibitory receptor PD-1, in the cells of lymphohematopoietic tissues Immunol Lett 2002 84 1 57 62 10.1016/S0165-2478(02)00142-6 12161284

[B29] Yamazaki T Akiba H Iwai H Matsuda H Aoki M Tanno Y Shin T Tsuchiya H Pardoll DM Okumura K Expression of programmed death 1 ligands by murine T cells and APC J Immunol 2002 169 10 5538 5545 12421930 10.4049/jimmunol.169.10.5538

[B30] Chen YB Mu CY Chen C Huang JA Association between single nucleotide polymorphism of PD-L1 gene and non-small cell lung cancer susceptibility in a Chinese population Asia Pac J Clin Oncol 2012 Doi:10.111/ajco.12307 10.1111/ajco.12037 23167931

[B31] Iwai Y Terawaki S Ikegawa M Okazaki T Honjo T PD-1 inhibits antiviral immunity at the effector phase in the liver J Exp Med 2003 198 1 39 50 10.1084/jem.20022235 12847136 PMC2196084

[B32] Ansari MJ Salama AD Chitnis T Smith RN Yagita H Akiba H Yamazaki T Azuma M Iwai H Khoury SJ The programmed death-1 (PD-1) pathway regulates autoimmune diabetes in nonobese diabetic (NOD) mice J Exp Med 2003 198 1 63 69 10.1084/jem.20022125 12847137 PMC2196083

[B33] Liang SC Latchman YE Buhlmann JE Tomczak MF Horwitz BH Freeman GJ Sharpe AH Regulation of PD-1, PD-L1, and PD-L2 expression during normal and autoimmune responses Eur J Immunol 2003 33 10 2706 2716 10.1002/eji.200324228 14515254

[B34] Wiendl H Mitsdoerffer M Schneider D Chen L Lochmuller H Melms A Weller M Human muscle cells express a B7-related molecule, B7-H1, with strong negative immune regulatory potential: a novel mechanism of counterbalancing the immune attack in idiopathic inflammatory myopathies FASEB J 2003 17 13 1892 1894 12923066 10.1096/fj.03-0039fje

[B35] Salama AD Chitnis T Imitola J Ansari MJ Akiba H Tushima F Azuma M Yagita H Sayegh MH Khoury SJ Critical role of the programmed death-1 (PD-1) pathway in regulation of experimental autoimmune encephalomyelitis J Exp Med 2003 198 1 71 78 10.1084/jem.20022119 12847138 PMC2196082

[B36] Dong H Strome SE Salomao DR Tamura H Hirano F Flies DB Roche PC Lu J Zhu G Tamada K Tumor-associated B7-H1 promotes T-cell apoptosis: a potential mechanism of immune evasion Nat Med 2002 8 8 793 800 12091876 10.1038/nm730

[B37] Chen X Liu S Wang L Zhang W Ji Y Ma X Clinical significance of B7-H1 (PD-L1) expression in human acute leukemia Cancer Biol Ther 2008 7 5 622 627 10.4161/cbt.7.5.5689 18756622

[B38] Mumprecht S Schurch C Schwaller J Solenthaler M Ochsenbein AF Programmed death 1 signaling on chronic myeloid leukemia-specific T cells results in T-cell exhaustion and disease progression Blood 2009 114 8 1528 1536 10.1182/blood-2008-09-179697 19420358

[B39] Ribas A Tumor immunotherapy directed at PD-1 N Engl J Med 2012 366 26 2517 2519 10.1056/NEJMe1205943 22658126

[B40] Matte-Martone C Venkatesan S Tan HS Athanasiadis I Chang J Pavisic J Shlomchik WD Graft-versus-leukemia (GVL) against mouse blast-crisis chronic myelogenous leukemia (BC-CML) and chronic-phase chronic myelogenous leukemia (CP-CML): shared mechanisms of T cell killing, but programmed death ligands render CP-CML and not BC-CML GVL resistant J Immunol 2011 187 4 1653 1663 10.4049/jimmunol.1100311 21768400 PMC3150287

[B41] Brusa D Serra S Coscia M Rossi D D’Arena G Laurenti L Jaksic O Fedele G Inghirami G Gaidano G The PD-1/PD-L1 axis contributes to T-cell dysfunction in chronic lymphocytic leukemia Haematologica 2013 98 6 953 963 10.3324/haematol.2012.077537 23300177 PMC3669453

[B42] Shimauchi T Kabashima K Nakashima D Sugita K Yamada Y Hino R Tokura Y Augmented expression of programmed death-1 in both neoplastic and non-neoplastic CD4+ T-cells in adult T-cell leukemia/lymphoma Int J Cancer 2007 121 12 2585 2590 10.1002/ijc.23042 17721918

[B43] Kozako T Yoshimitsu M Fujiwara H Masamoto I Horai S White Y Akimoto M Suzuki S Matsushita K Uozumi K PD-1/PD-L1 expression in human T-cell leukemia virus type 1 carriers and adult T-cell leukemia/lymphoma patients Leukemia 2009 23 2 375 382 10.1038/leu.2008.272 18830259

[B44] Yamamoto R Nishikori M Kitawaki T Sakai T Hishizawa M Tashima M Kondo T Ohmori K Kurata M Hayashi T PD-1-PD-1 ligand interaction contributes to immunosuppressive microenvironment of Hodgkin lymphoma Blood 2008 111 6 3220 3224 10.1182/blood-2007-05-085159 18203952

[B45] Zhou Q Munger ME Veenstra RG Weigel BJ Hirashima M Munn DH Murphy WJ Azuma M Anderson AC Kuchroo VK Coexpression of Tim-3 and PD-1 identifies a CD8+ T-cell exhaustion phenotype in mice with disseminated acute myelogenous leukemia Blood 2011 117 17 4501 4510 10.1182/blood-2010-10-310425 21385853 PMC3099570

[B46] Liechtenstein T Dufait I Bricogne C Lanna A Pen J Breckpot K Escors D PD-L1/PD-1 Co-stimulation, a brake for T cell activation and a T cell differentiation signal J Clin Cell Immunol 2012 S12 10.4172/2155-9899.S12-006 PMC3605779 23525238

[B47] Christiansson L Soderlund S Svensson E Mustjoki S Bengtsson M Simonsson B Olsson-Stromberg U Loskog AS Increased level of myeloid-derived suppressor cells, programmed death receptor ligand 1/programmed death receptor 1, and soluble CD25 in Sokal high risk chronic myeloid leukemia PLoS One 2013 8 1 e55818 10.1371/journal.pone.0055818 23383287 PMC3561335

[B48] Peng W Lizee G Hwu P Blockade of the PD-1 pathway enhances the efficacy of adoptive cell therapy against cancer Oncoimmunology 2013 2 2 e22691 10.4161/onci.22691 23524510 PMC3601154

[B49] Brahmer JR Tykodi SS Chow LQ Hwu WJ Topalian SL Hwu P Drake CG Camacho LH Kauh J Odunsi K Safety and activity of anti-PD-L1 antibody in patients with advanced cancer N Engl J Med 2012 366 26 2455 2465 10.1056/NEJMoa1200694 22658128 PMC3563263

[B50] Chen BJ Chapuy B Ouyang J Sun HH Roemer MG Xu ML Yu H Fletcher CD Freeman GJ Shipp MA PD-L1 expression is characteristic of a subset of aggressive B-cell lymphomas and virus-associated malignancies Clin Cancer Res 2013 19 13 3462 3473 10.1158/1078-0432.CCR-13-0855 23674495 PMC4102335

[B51] Andorsky DJ Yamada RE Said J Pinkus GS Betting DJ Timmerman JM Programmed death ligand 1 is expressed by non-hodgkin lymphomas and inhibits the activity of tumor-associated T cells Clin Cancer Res 2011 17 13 4232 4244 10.1158/1078-0432.CCR-10-2660 21540239

[B52] Berger R Rotem-Yehudar R Slama G Landes S Kneller A Leiba M Koren-Michowitz M Shimoni A Nagler A Phase I safety and pharmacokinetic study of CT-011, a humanized antibody interacting with PD-1, in patients with advanced hematologic malignancies Clin Cancer Res 2008 14 10 3044 3051 10.1158/1078-0432.CCR-07-4079 18483370

[B53] Shi L Chen S Lu Y Wang X Xu L Zhang F Yang L Wu X Li B Li Y Changes in the MALT1-A20-NF-kappaB expression pattern may be related to T cell dysfunction in AML Cancer Cell Int 2013 13 1 37 10.1186/1475-2867-13-37 23627638 PMC3641943

